# Dietary L-Citrulline Supplementation Promotes Rumen Development and Modulates the Microbiota–Metabolome Axis in Suckling Hu Lambs

**DOI:** 10.3390/ani16111728

**Published:** 2026-06-04

**Authors:** Zhen Tang, Shuoyi Zhang, Peiyao Xu, Honggang Tang, Weiyi Gao, Ying Cao, Ruobing Zhai, Kaixu Chen

**Affiliations:** Xinjiang Key Laboratory of Herbivore Nutrition for Meat & Milk, College of Animal Science, Xinjiang Agricultural University, Urumqi 830052, China; 19882959057@163.com (Z.T.); 15233020401@163.com (S.Z.); xpy269271@126.com (P.X.); 15576409357@163.com (H.T.); 13116043510@163.com (W.G.); 18997775661@163.com (Y.C.); 18167989138@163.com (R.Z.)

**Keywords:** L-citrulline, suckling lamb, rumen development, volatile fatty acids, 16S rRNA sequencing, untargeted metabolomics

## Abstract

The suckling phase is the critical window for rumen functional maturation, yet amino-acid-based interventions tailored to this stage remain scarce. L-citrulline (L-cit) bypasses hepatic first-pass metabolism, is converted to L-arginine peripherally, and resists ruminal microbial degradation, making it a candidate functional additive for early-life ruminants. This study evaluated whether dietary L-cit at 2 g·lamb^−1^·d^−1^ would improve rumen development and metabolic function in suckling Hu lambs. Twenty male Hu lambs were randomly assigned to a control (CON) or L-cit group (*n =* 10/group) and reared for 45 d (3 d adaptation + 42 d treatment). Growth and starter intake were assessed in all lambs; six lambs per group (*n* = 6) were subsequently slaughtered for rumen morphometry, gas chromatography–flame ionization detection (GC-FID) volatile fatty acid (VFA) quantification, 16S rRNA gene sequencing, and liquid chromatography–mass spectrometry (LC-MS) untargeted metabolomics. L-cit increased average daily starter intake by 25.96% (*p* = 0.036) and produced a 20.00% numerical but non-significant increase in average daily gain (ADG) (*p* = 0.203; Cohen’s d = 0.58). Rumen weight, volume, and papillary length, width, density, and epithelial thickness were all elevated (*p* < 0.05), whereas muscular thickness was unaffected (*p* = 0.162). Total VFA, acetate, propionate (+37.64%, *p* < 0.001), and butyrate were higher in the L-cit group; the molar proportion of propionate rose from 21.41% to 24.75%, and the acetate-to-propionate ratio declined from 2.90 to 2.44 (*p* = 0.005). Microbial richness (Chao1, Observed species) increased without altered evenness, and linear discriminant analysis effect size (LEfSe) identified L-cit-driven enrichment of propionate-generating and fiber-degrading genera, including *Prevotellaceae_UCG-004*, *Ruminobacter*, and the *NK4A214_group*. Of 539 differential metabolites (147 of which were annotated to the Kyoto Encyclopedia of Genes and Genomes (KEGG) database), KEGG enrichment highlighted linoleic acid metabolism and purine metabolism as the biologically interpretable targets. Microbiota–metabolite correlations linked L-cit-enriched genera to up-regulated metabolites such as adenine. Dietary L-cit at 2 g·lamb^−1^·d^−1^ enhances starter intake, promotes rumen epithelial development, promotes a shift toward enhanced propiogenic fermentation within an acetate-dominant profile, and remodels the microbiota–metabolome axis, supporting its application as a functional additive during the suckling phase of ruminants. Because epithelial barrier integrity, oxidative stress, and inflammatory markers were not directly measured, these findings should be interpreted as morphological and association-based evidence, and further functional validation is required.

## Simple Summary

Young lambs that still drink milk often have underdeveloped stomachs, which limits how well they can digest solid food and grow during their first weeks of life. Because of growing concerns about antibiotic use in livestock, farmers and scientists are searching for natural feed supplements that can help young animals develop more quickly and stay healthy. This study tested whether adding a small daily dose of L-citrulline, a natural amino acid that the body converts into another helpful amino acid called arginine, could support stomach development in suckling lambs. Twenty male Hu lambs were divided into two equal groups and reared for forty-five days. Lambs receiving L-citrulline ate about twenty-six percent more solid starter feed than untreated lambs and developed larger, heavier stomachs with thicker tissue and longer finger-like surface projections that improve nutrient absorption. The supplement also increased helpful fermentation acids that fuel lamb growth and encouraged a richer community of beneficial microbes inside the stomach. Together, these findings suggest that L-citrulline may be a promising nutritional strategy to support early rumen development and the transition to solid feed in suckling lambs. However, epithelial barrier integrity, oxidative stress, inflammatory markers, and disease resistance were not directly assessed; therefore, further studies are needed before drawing conclusions about its effects on gut barrier function, immune status, or antibiotic reduction.

## 1. Introduction

In intensive production systems, suckling lambs commonly face challenges including underdeveloped rumens, imbalanced nutrient intake, and increased exposure to pathogens; consequently, improving their health status and growth performance constitutes an indispensable component of the sustainable development of the meat sheep industry [1,2]. Although antibiotic growth promoters (AGPs) have long been employed to improve ruminant productivity, the proliferation of antimicrobial resistance, residues in animal-source foods, and ecological risks have led to stringent restrictions on their use [3]. Within the broader context of developing AGP alternatives, functional amino acids, which possess both nutritional and physiological regulatory properties, have attracted increasing attention in ruminant nutrition [4].

L-citrulline (L-cit) is a non-protein α-amino acid synthesized in mammals by intestinal epithelial cells from glutamine and glutamate as precursors and subsequently converted into L-arginine in the kidney, thereby bypassing the hepatic first-pass uptake of L-arginine [5,6]. Through its conversion to L-arginine, L-cit participates in the arginine–nitric oxide (NO) pathway and the mammalian target of rapamycin (mTOR) signaling pathway, both of which are implicated in gastrointestinal epithelial development [4]. In ruminants, an in vitro incubation study confirmed that exogenous L-cit was virtually undegraded by adult ovine rumen microbiota, indicating that L-cit may serve as a rumen-protected L-arginine precursor [7]. The previous research results of our research group indicated that supplementing 10 g/d L-cit could optimize the rumen microbiota structure of Hu sheep ewes, increase the levels of reproductive hormones and antioxidant capacity in plasma, and regulate the proportion of rumen VFA [8]. Existing evidence, however, has been largely confined to adult animals; whether L-cit retains detectable bioavailability and corresponding physiological functional value in suckling lambs—whose rumen microbial metabolic system and epithelial barrier have not yet attained functional maturity—remains empirically unexamined and warrants systematic elucidation.

The suckling period represents a critical window during which the digestive physiology of ruminants shifts from a pseudo-monogastric state toward a functional rumen. During this stage, microbial colonization, volatile fatty acid production, starter feed intake, and rumen epithelial development are closely coordinated and collectively determine subsequent ruminal fermentation capacity and feed utilization efficiency [9,10,11]. Starter feed intake is the principal environmental factor triggering microbial remodeling and epithelial development [12,13]; accordingly, nutritional strategies capable of enhancing starter intake and stimulating epithelial development and microbial metabolism in young ruminants carry substantial practical importance for fully realizing their early-life productive potential [1]. Given the aforementioned roles of L-cit in the Arg–NO and mTOR pathways, which have been shown to promote intestinal villus development and feed-intake regulation in monogastric neonates [4], L-cit may exert analogous benefits on rumen development and starter intake in suckling lambs.

The rumen microbial ecosystem is composed of anaerobic bacteria, archaea, protozoa, fungi, and bacteriophages, whose metabolic products—volatile fatty acids (VFA)—provide approximately 70% of the energy requirements of ruminants [11,14]. Multi-omics approaches integrating 16S rRNA high-throughput sequencing and LC-MS-based untargeted metabolomics have become the predominant methodology for resolving the rumen microbiota–metabolite axis and its interactions with host phenotypes [15,16,17]. Nevertheless, studies addressing the integrative effects of direct L-cit supplementation on rumen development, fermentation parameters, microbial community structure, and metabolic profile in suckling lambs remain markedly limited.

On the basis of the foregoing background and our group’s previous work, we hypothesized that supplementing suckling lambs with L-cit at 2 g·d^−1^ per lamb would synergistically promote early rumen development. This dose was selected on the basis of three converging lines of evidence. First, a preliminary dose–response study conducted by our research group in Hu sheep identified 2 g·d^−1^ as the minimum effective dose that improved rumen fermentation parameters without observable adverse effects. Second, 2 g·d^−1^ corresponds to approximately 0.22 g·kg^−1^ BW·d^−1^ at the average body weight of suckling lambs during the experimental period, which is allometrically consistent with the 0.21 g·kg^−1^ BW·d^−1^ effective dose validated in adult Hu ewes by Liu et al. (2025) [8]. Third, the resulting body-weight-normalized dose falls within the 0.10–0.30 g·kg^−1^ BW·d^−1^ range reported as safe and efficacious for L-cit supplementation in human neonates [18] and neonatal piglets [19], suggesting cross-species convergence around this dose window for L-cit as an arginine precursor in early-life mammals. Using male Hu lambs as the model, we integrated measurements of starter intake, rumen histomorphology, GC-FID-based VFA quantification, 16S rRNA high-throughput sequencing, and LC-MS untargeted metabolomics to systematically evaluate the comprehensive effects of L-cit on rumen development, fermentation parameters, microbial community structure, metabolic profile, and microbiota–metabolite association networks in suckling lambs, thereby providing preliminary evidence for the application of L-cit as a functional feed additive for ruminants during the suckling phase.

## 2. Materials and Methods

### 2.1. Ethics Statement

All experimental procedures were conducted in strict accordance with the guidelines for animal welfare and the use of laboratory animals established by the Institutional Animal Care and Use Committee of Xinjiang Agricultural University, and the protocol was reviewed and approved by the same committee (Approval No. 2023038, Approval Date: 14 May 2023). The experimental design and reporting fully complied with the ARRIVE 2.0 guidelines (Animal Research: Reporting of In Vivo Experiments).

### 2.2. Experimental Materials

L-citrulline (L-cit; purity ≥ 99%, HPLC grade) was purchased from Wuhan Jixin Yibang Biotechnology Co., Ltd. (Wuhan, China). Hu sheep (*Ovis aries*) male lambs used in this trial were provided by Xinjiang Shangpin Meiyang Technology Co., Ltd. (Urumqi, China). The milk replacer, starter feed, and conventional premix ingredients were supplied by Xinjiang Huikang Animal Husbandry Technology Co., Ltd. (Urumqi, China).

### 2.3. Experimental Animals and Experimental Design

A completely randomized single-factor design was adopted, and the trial was carried out at Xinjiang Huikang Animal Husbandry Technology Co., Ltd. from June to August 2024. Twenty healthy male lambs born to Hu ewes of identical parity (parities 2–3), with similar parturition dates (within ±3 d), comparable maternal body condition scores, and good health status were selected. The randomization sequence was computer-generated prior to the start of the trial using the RAND function in Microsoft Excel 2019. Lambs were first ranked by initial body weight (BW) and then assigned to either the control group (CON) or the L-citrulline supplementation group (L-cit) via stratified block randomization (block size = 2), with initial BW as the stratification variable, to ensure comparable baseline weights between groups. To ensure allocation concealment, the group assignments were enclosed in sequentially numbered, opaque, sealed envelopes prepared by one investigator (K.C.) who was not involved in animal enrollment, daily management, or sample collection. Envelopes were opened only after each lamb had been formally enrolled and its baseline BW recorded. Sample collection and laboratory analyses were performed by personnel blinded to group allocation, ensuring a single-blind protocol.

Lambs were nursed by their dams from days 1–3 to ensure adequate colostrum intake. From day 4 onwards, lambs were separated from their dams and transferred to artificial rearing pens for milk-replacer gavage feeding. Each lamb was housed in an individual pen (1.2 m × 1.0 m) equipped with a dedicated starter-feed trough and water bucket, ensuring that daily starter feed intake could be recorded on an individual-lamb basis.Lambs in the L-cit group received fixed daily dose of 2 g·lamb^−1^·d^−1^ L-citrulline per lamb, divided equally into three portions and administered together with the milk replacer at 08:00, 14:00, and 20:00. Lambs in the CON group received an equal volume of milk replacer without L-cit supplementation. The selected dose was based on a preliminary dose–response study previously conducted by our research group in Hu sheep, and corresponds to approximately 0.22 g·kg^−1^ BW·d^−1^ at the average body weight of suckling lambs during the experimental period—consistent with the 0.21 g·kg^−1^ BW·d^−1^ effective dose validated in adult Hu ewes by Liu et al. (2025) [8].

At the end of the 42 d treatment period (day 48), six lambs per group (*n* = 6) with body weights closest to the respective group mean were selected for slaughter and post-mortem sample collection; the remaining four lambs per group were not subjected to slaughter and were maintained under standard rearing conditions. The selected lambs were fasted for 12 h with free access to water before slaughter. Accordingly, all in vivo measurements (initial BW, weekly BW, ADG, and starter-feed intake) were performed on all 20 lambs (*n* = 10 per group), whereas all post-mortem analyses (rumen developmental parameters, VFA profile, 16S rRNA gene sequencing, and untargeted metabolomic analysis) were performed on the 12 selected lambs (*n* = 6 per group).

### 2.4. Feeding Management and Dietary Composition

The daily milk-replacer allowance was calculated as 2% of initial BW (dry matter, DM, basis) and reconstituted with warm water at 50 °C at a feed-to-water ratio of 1∶5 to prepare a liquid milk; once the temperature had cooled to 38–40 °C, it was administered by gavage. The daily allowance was recalculated weekly based on fasted BW. During the entire process of gavage feeding, pay close attention to the lamb’s breathing and swallowing response to prevent the risk of aspiration cough and choking. For the L-cit group, the daily L-cit dose (2 g·lamb^−1^·d^−1^) was equally divided into three portions (approximately 0.67 g per feeding), weighed, and added to the liquid milk prior to feeding; complete dissolution was achieved by mixing on a vortex shaker (Vortex-Genie 2, Scientific Industries, Bohemia, NY, USA) for 30 s. Three feedings were administered daily at 08:00, 14:00, and 20:00.

Beginning at 15 days of age, starter feed and clean drinking water were provided ad libitum in the rearing pens. Pen temperature was maintained at 18–24 °C with relative humidity of 55–65%; natural lighting was supplemented with artificial illumination to provide a 12 h photoperiod, with ventilation maintained for ≥4 h daily. Bedding was replaced every 3 d, and routine disinfection of pens was performed. Lambs were vaccinated according to the locally recommended immunization protocol. Throughout the experimental period, the health status of each lamb (feed intake, respiration, fecal characteristics, and coat condition) was observed and recorded daily; no lamb mortality or severe illness occurred during the trial. The ingredient composition and nutrient levels of the milk replacer and starter feed are presented in Table 1 and Table 2, respectively.

### 2.5. Sample Collection

At the end of the treatment period (day 48), following 12 h of fasting after the final feeding, the six selected lambs per group (*n =* 6) were slaughtered by exsanguination. Immediately after slaughter, the abdominal cavity was opened and the rumen was excised. The rumen was incised along the dorsal–ventral junction of the ventral sac, and after thorough mixing of rumen contents, samples were partitioned as follows: (i) 50 mL of rumen fluid was filtered through four layers of sterile gauze, aliquoted into 2 mL sterile cryotubes, immediately snap-frozen in liquid nitrogen, and transferred to a −80 °C freezer for subsequent 16S rRNA gene sequencing and untargeted metabolomic analyses; (ii) 10 mL of rumen fluid was added to a sampling tube containing 2 mL of 25% metaphosphoric acid solution (containing 25 mmol·L^−1^ 2-ethylbutyric acid as internal standard), vortex-mixed, allowed to stand at 4 °C for 30 min, and centrifuged at 12,000× *g* for 15 min at 4 °C. The supernatant was collected and stored at −80 °C until volatile fatty acid (VFA) determination.

### 2.6. Measurements and Analytical Methods

#### 2.6.1. Growth Performance and Feed Intake

Lambs were weighed weekly (every Monday) before morning feeding using an electronic platform scale with 0.01 kg precision (YH-T7, Shanghai Yingzhan Industrial, Shanghai, China) following overnight fasting. Initial BW (day 4), final BW (day 48), total weight gain (TWG), and average daily gain (ADG) were calculated. From day 15 onwards, residual starter feed in each pen from the previous day was weighed and recorded daily at 07:30. Daily actual feed intake (as-fed basis) was calculated as the difference between feed offered and feed refused, and was subsequently converted to dry matter intake (DMI, g·d^−1^) on the basis of the DM content of the starter feed (83.16%). The age at first voluntary starter intake and cumulative starter intake from day 15 to day 48 were also recorded for each lamb.

#### 2.6.2. Determination of Rumen Developmental Parameters

Rumen developmental parameters were determined in the slaughter-sampled lambs (*n* = 6 per group). After slaughter, the rumen was completely excised, the contents were emptied, and the rumen wall was rinsed with 0.9% physiological saline. After blotting with filter paper, the empty rumen tissue was weighed using an electronic balance with 0.1 g precision (ME2002, Mettler-Toledo, Greifensee, Switzerland), and the rumen weight to live weight ratio (g·kg^−1^) was calculated. Rumen volume was measured by the water-filling method: after ligating all openings of the rumen, warm water at 38 °C was injected until the rumen wall was fully extended but not distended, and the volume of water injected (mL) was recorded as rumen volume.

Tissue blocks of approximately 2 × 2 cm^2^ were excised from the middle of the ventral sac, fixed in 4% paraformaldehyde for 24 h, dehydrated through a graded ethanol series, cleared in xylene, embedded in paraffin, sectioned at 5 μm thickness, and stained with hematoxylin and eosin (H&E). Sections were observed under a light microscope (CX23, Olympus, Japan), and rumen papilla length, papilla width (at the base), total epithelial thickness, and muscle layer thickness were measured using Image-Pro Plus 6.0 (Media Cybernetics, Rockville, MD, USA). At least 10 intact papillae were measured per lamb, and the mean values were used as individual data. Papilla density was determined by direct counting within a 1 × 1 cm^2^ marked area.

#### 2.6.3. Determination of Rumen Volatile Fatty Acids (VFAs)

VFA determinations were performed on rumen-fluid samples obtained from the slaughter-sampled lambs (*n* = 6 per group). Rumen-fluid supernatant was filtered through a 0.22 μm membrane and analyzed for VFAs by gas chromatography–flame ionization detection (GC-FID; Agilent 7890B, Santa Clara, CA, USA). The chromatographic column was an HP-FFAP capillary column (30 m × 0.25 mm × 0.25 μm; Agilent, USA). The injector and detector temperatures were set at 250 °C and 280 °C, respectively. The temperature program was as follows: an initial temperature of 100 °C held for 1 min, increased to 180 °C at 10 °C·min^−1^ and held for 3 min, then increased to 220 °C at 20 °C·min^−1^ and held for 2 min. High-purity nitrogen (≥99.999%) was used as the carrier gas at a flow rate of 1.0 mL·min^−1^, with a split ratio of 10∶1 and an injection volume of 1 μL. Acetate, propionate, butyrate, isobutyrate, valerate, isovalerate, and 2-methylbutyrate were quantified using the external-standard method combined with internal-standard correction (2-ethylbutyric acid). Standards were obtained from Sigma-Aldrich (Sigma-Aldrich, St. Louis, MO, USA), with calibration curves spanning 0.1–20 mmol·L^−1^ and R^2^ ≥ 0.999 for all VFAs. Results were expressed both as absolute concentrations (mmol·L^−1^) and as molar percentages (mol%) of total VFAs (TVFAs), and the acetate-to-propionate ratio (A/P) was calculated.

#### 2.6.4. 16S rRNA Gene Sequencing of the Rumen Microbiota

Rumen-fluid samples from the slaughter-sampled lambs (*n* = 6 per group; 12 samples in total) were sent to Beijing Novogene Co., Ltd. (Beijing, China) for 16S rRNA gene sequencing. Genomic DNA was extracted using the cetyltrimethylammonium bromide (CTAB) method. DNA purity and concentration were verified using a NanoDrop 2000 spectrophotometer (Thermo Fisher Scientific, Waltham, MA, USA) and 1% agarose gel electrophoresis. The V3–V4 hypervariable region of the 16S rRNA gene was amplified using the specific primers 341F (5′-CCTAYGGGRBGCASCAG-3′) and 806R (5′-GGACTACHVGGGTWTCTAAT-3′). The PCR reaction system (30 μL) consisted of 15 μL Phusion^®^ High-Fidelity PCR Master Mix (New England Biolabs, Ipswich, MA, USA), 1 μL of each forward and reverse primer (0.2 μmol·L^−1^), and 10 ng of genomic DNA template, with the volume adjusted to 30 μL using sterile deionized water. The PCR program was as follows: pre-denaturation at 98 °C for 1 min; 30 cycles of denaturation at 98 °C for 10 s, annealing at 50 °C for 30 s, and extension at 72 °C for 30 s; followed by a final extension at 72 °C for 5 min. The PCR products were verified by 2% agarose gel electrophoresis, purified using AMPure XP magnetic beads (Beckman Coulter, Brea, CA, USA), and pooled in equimolar amounts. Libraries were constructed using the TruSeq^®^ DNA PCR-Free Library Preparation Kit (Illumina, San Diego, CA, USA), and library quality was assessed using a Qubit 4.0 fluorometer (Thermo Fisher Scientific, USA) and qPCR (Applied Biosystems 7500, Thermo Fisher Scientific, Waltham, MA, USA). Sequencing was performed on the Illumina NovaSeq 6000 platform with paired-end reads of 250 bp (PE250, 2 × 250 bp).

Raw sequencing reads were processed by trimming primer and adapter sequences using Cutadapt v1.9.1, and the paired-end reads were merged using FLASH v1.2.11 to obtain raw tags. Strict quality filtering (mean Q ≥ 20, length ≥ 200 bp) was performed using fastp v0.23.1 to obtain clean tags. Chimeras were identified and removed by alignment to the SILVA v138.1 database using the UCHIME algorithm (v7.0), yielding effective tags. Operational taxonomic units (OTUs) were clustered using UPARSE v7.0.1001 at a 97% sequence-similarity threshold. To enhance the robustness of the results, amplicon sequence variants (ASVs) were also constructed in parallel using DADA2 (QIIME2 v2023.9), and parallel validation of the principal α- and β-diversity outcomes was performed; the trends obtained were consistent across both pipelines. Representative sequences were taxonomically annotated against the SILVA v138.1 database.

Sequencing depth normalization was performed prior to all diversity analyses. For α-diversity, all 12 samples were rarefied (subsampled without replacement) to the minimum observed sequencing depth across the dataset (78,436 effective tags per sample) using the feature-table rarefy plugin in QIIME2 v2023.9, with a fixed random seed (seed = 42) to ensure reproducibility. The adequacy of this rarefied depth was confirmed by two criteria: (i) rarefaction curves for all samples reached an asymptotic plateau, indicating that additional sequencing would recover negligible additional diversity; and (ii) Good’s coverage exceeded 0.998 in all rarefied samples, confirming near-complete capture of the microbial community. α-Diversity indices (Chao1, Observed species, Shannon, Simpson, and Good’s coverage) were calculated from the rarefied OTU table using the QIIME2 feature-table plugin. For β-diversity, two complementary normalization approaches were applied to assess the sensitivity of community-level comparisons to the normalization method. The primary analysis used the same rarefied OTU table (78,436 tags per sample) to compute Bray–Curtis distance matrices and perform principal coordinate analysis (PCoA). As a supplementary validation, total-sum scaling (TSS; i.e., conversion to relative abundances) was applied to the unrarefied OTU table, and Bray–Curtis-based PCoA was recalculated; ordination patterns and PERMANOVA outcomes were consistent between the two approaches, confirming that the observed inter-group differences were not artifacts of the normalization strategy. The significance of intergroup β-diversity differences was tested using PERMANOVA (999 permutations); homogeneity of multivariate dispersion was evaluated using the PERMDISP test. For differential abundance analysis (LEfSe), genus-level relative abundances derived from TSS normalization were used as input, consistent with the compositional-data requirements of the algorithm. The α level for the non-parametric Kruskal–Wallis test was set at 0.05, and the LDA score threshold was set at >3.5 to control false positives; both raw *p* values and LDA scores are reported. The parallel ASV-based pipeline (DADA2, QIIME2 v2023.9) was rarefied to the same depth threshold, and the principal α- and β-diversity outcomes were consistent with those obtained from the OTU-based pipeline, supporting the robustness of the results to both clustering method and normalization approach.

#### 2.6.5. Untargeted Metabolomic Analysis of the Rumen Fluid

Untargeted metabolomic analysis was performed on rumen-fluid samples from the slaughter-sampled lambs (*n* = 6 per group; 12 samples in total).

(1) Sample pretreatment

A 100 μL aliquot of rumen fluid was placed into a 1.5 mL centrifuge tube, and 400 μL of pre-cooled (−20 °C) 80% methanol aqueous solution containing an isotopic internal standard (L-2-chlorophenylalanine, 1 μg·mL^−1^) was added. After vortexing for 30 s and incubation on ice for 5 min, the mixture was centrifuged at 15,000× *g* for 20 min at 4 °C. Subsequently, 200 μL of the supernatant was transferred to a sample vial containing 200 μL of LC-MS-grade water (final methanol concentration of 53%), and the mixture was centrifuged again at 15,000× *g* for 20 min at 4 °C; the resulting supernatant was used for LC-MS analysis. A quality control (QC) sample (prepared by mixing equal volumes of supernatants from all samples) was inserted every 10 samples to monitor instrument stability and data quality.

(2) LC-MS parameters

Analyses were performed on a Vanquish UHPLC system (Thermo Fisher Scientific GmbH, Dreieich, Germany) coupled to a Q Exactive™ HF-X high-resolution mass spectrometer (Thermo Fisher Scientific GmbH, Dreieich, Germany). The chromatographic column was a Hypersil Gold C_18_ column (100 mm × 2.1 mm, 1.9 μm; Thermo Fisher Scientific, Waltham, MA, USA) maintained at 40 °C, with a flow rate of 0.2 mL·min^−1^ and an injection volume of 2 μL. In positive ion mode, mobile phase A consisted of 0.1% formic acid and 95% acetonitrile in 10 mmol·L^−1^ aqueous ammonium acetate, and mobile phase B consisted of 0.1% formic acid and 50% acetonitrile in 10 mmol·L^−1^ aqueous ammonium acetate. In negative ion mode, mobile phase A consisted of 95% acetonitrile in 10 mmol·L^−1^ aqueous ammonium acetate, and mobile phase B consisted of 50% acetonitrile in 10 mmol·L^−1^ aqueous ammonium acetate. The gradient elution program was as follows: 0–12 min, B 2% → 100%; 12–14 min, B 100%; 14–17 min, B 100% → 2%.

The mass-spectrometric parameters were as follows: scan range, m/z 100–1500; spray voltage, +3.5 kV (positive mode) and −3.0 kV (negative mode); sheath gas flow rate, 241 kPa (35 psi); auxiliary gas flow rate, 10 L·min^−1^; ion transfer tube temperature, 320 °C; auxiliary gas heater temperature, 350 °C; and S-lens RF level, 60. Data-dependent MS/MS acquisition (DDA) mode was used, with full-scan and MS^2^ resolutions of 70,000 and 17,500, respectively.

(3) Data processing and metabolite identification

Raw files (.raw) were converted to mzXML format using ProteoWizard v3.0, and peak extraction, retention-time correction, and peak alignment (mass deviation ≤ 10 ppm; retention-time deviation ≤ 0.15 min) were performed using XCMS v3.16. Metabolites were identified by matching exact molecular mass (deviation ≤ 10 ppm), MS/MS fragmentation patterns, and retention times against the mzCloud, mzVault, HMDB v5.0, KEGG, and LipidMaps databases. The confidence of metabolite identification was classified according to the Metabolomics Standards Initiative (MSI) into Level 1 (validated against authentic standards), Level 2 (database matching), and Level 3 (chemical-class identification). After background-ion subtraction (using blank controls), raw quantitative values were normalized batch-wise according to the following equation:

Relative Peak Areaij = [xij/Σk = 1m xik] × Σk = 1m × QC1,k(1)

where xij is the raw peak area of metabolite j in sample i, m is the total number of metabolites in that sample, and Σ × QC1,k is the sum of all metabolite peak areas in the first QC sample. After normalization, metabolites with a coefficient of variation (CV) > 30% in the QC samples were removed to ensure data stability.

(4) Metabolomic statistical analysis

Data transformation (log_2_ transformation followed by Pareto centering) was performed using metaX v1.4.16, and principal component analysis (PCA) and orthogonal partial least squares discriminant analysis (OPLS-DA) were conducted in R v4.3.1. The robustness of the OPLS-DA model was evaluated by 7-fold cross-validation and 200-permutation testing; R^2^X, R^2^Y, Q^2^, and the Q^2^ intercept of the permutation test were reported (acceptance criteria: R^2^Y > 0.5, Q^2^ > 0.4, permutation Q^2^ intercept < 0.05). The screening of differential metabolites was conducted based on the following three criteria: variable importance in projection (VIP) > 1, *p* value < 0.05, and fold change (FC) > 1.5 or <2/3 (i.e., FC < 0.667). The volcano plot was drawn using the ggplot2 package (Version 4.0.3) in R software (Version 4.3.1), integrating the VIP value, log_2_(FC), and −log_10_ (*p* value) of each metabolite to visually identify candidate metabolites with statistical significance and biological effect intensity. Functional annotation and pathway enrichment analysis were carried out based on the KEGG database. The significance of pathway enrichment was determined by hypergeometric test, that is, when the proportion of significantly differentially expressed metabolites in the target pathway (x/n) was significantly higher than the theoretical proportion of the pathway in the entire metabolite set (y/N), the pathway was considered to be significantly enriched (*p* < 0.05). All bioinformatics analyses were performed on the Novogene Cloud Analysis Platform (NovoMagic Cloud, https://magic.novogene.com, accessed on 15 January 2026).

### 2.7. Correlation Analysis

Because 16S rRNA relative-abundance data are compositional in nature, genus-level relative abundances were first transformed using the centered log-ratio (CLR) transformation. Spearman’s rank correlation coefficients were then calculated to evaluate associations between rumen microbial taxa (differential genera identified by LEfSe and core dominant genera) and differential metabolites. Correlation heatmaps were generated using the Metware Cloud platform (https://cloud.metware.cn, accessed on 15 January 2026), with *p* values (denoted as *, **, ***) used to indicate significance levels.

### 2.8. Statistical Analysis

Experimental data were preliminarily organized using Microsoft Excel 2019, and statistical analyses were performed using SPSS 27.0 (IBM Corp., Armonk, NY, USA) and GraphPad Prism 10.0.0 (GraphPad Software, Boston, MA, USA); high-dimensional omics data analyses and visualization were performed in R v4.3.1.

Sample sizes differed between in vivo and post-mortem analyses. In vivo measurements (initial BW, final BW, weekly BW, TWG, ADG, and starter-feed intake) were performed on all 20 lambs (*n* = 10 per group), whereas post-mortem analyses (rumen developmental parameters, VFA profile, 16S rRNA gene sequencing, and untargeted metabolomic analysis) were performed on the 12 selected lambs (*n* = 6 per group). The sample size for each analysis is explicitly indicated in the corresponding tables and figures.

For continuous variables, normality of distribution was assessed using the Shapiro–Wilk test, and homogeneity of variance was tested using Levene’s test. Data meeting both assumptions were analyzed using Student’s independent-sample *t*-test; otherwise, the Mann–Whitney U test was applied. For growth performance variables (final BW, TWG, and ADG), analysis of covariance (ANCOVA) was performed with initial BW as the covariate to adjust for any baseline differences, and least squares means (LSM ± SEM) adjusted for the covariate are reported. Given the repeated weekly BW measurements, weight changes over time were analyzed using a linear mixed model (LMM), with treatment, time, and their interaction set as fixed effects and individual lamb as a random effect. The covariance structure was selected from first-order autoregressive (AR1), compound symmetry (CS), and unstructured (UN) on the basis of the Akaike information criterion (AIC). The UN structure failed to converge owing to overparameterization relative to the sample size (28 covariance parameters vs. 20 subjects) and was therefore excluded. The AR1 structure provided the best fit (AIC = 486.3 vs. CS: AIC = 493.8; ΔAIC = 7.5) and was adopted for the final model. Under the AR1 structure, the time effect was highly significant (F(6, 108) = 314.26, *p* < 0.001), confirming the expected progressive weight gain over the experimental period. The treatment × time interaction was non-significant (F(6, 108) = 1.58, *p* = 0.160), indicating that the two groups exhibited largely parallel growth trajectories despite the numerically greater weight gain in the L-cit group, which is consistent with the moderate but non-significant ADG difference reported above (Cohen’s d = 0.58, *p* = 0.203)

All experimental data are presented as the mean ± standard deviation (mean ± SD); where applicable, effect sizes (Cohen’s d) and 95% confidence intervals are also reported. For all analyses, *p* values are reported. Differences were considered statistically significant at *p* < 0.05, and 0.05 ≤ *p* < 0.10 was considered to indicate a tendency. The statistical model can be expressed as follows:

Yij = μ + Ti + βXij + εij(2)

where Yij is the dependent variable (e.g., ADG or VFA concentration), μ is the overall mean, Ti is the fixed effect of treatment group (i = CON, L-cit), Xij is the covariate (initial BW), β is the regression coefficient associated with the covariate, and εij is the residual term, where εij ~ N(0, σ^2^).

Retrospective (observed) power analyses were performed using G*Power 3.1.9.7 (Heinrich-Heine-Universität Düsseldorf, Germany) [20] to evaluate whether the achieved sample sizes provided adequate statistical power for the key outcome variables. All power calculations were based on a two-tailed independent-samples *t*-test at α = 0.05, using the observed effect sizes (Cohen’s d) from the present study. Results are reported in Section 4.7 and Supplementary Table S3.

## 3. Results

### 3.1. Effects of Dietary L-Cit Supplementation on Lamb Growth Performance

The effects of dietary L-cit supplementation on the growth performance of suckling Hu lambs are presented in Table 3. Initial body weight (BW) was comparable between the two groups (CON: 5.19 ± 0.96 kg vs. L-cit: 5.29 ± 0.69 kg; *p* = 0.787), confirming the appropriateness of the random allocation. After the 42 d feeding trial, final BW, total weight gain (TWG), and average daily gain (ADG) of the L-cit group were numerically increased by 6.88%, 16.90%, and 20.00%, respectively, compared with the CON group. However, after analysis of covariance (ANCOVA, with initial BW as a covariate), none of these differences reached statistical significance (final BW adjusted LSM: 13.21 vs. 12.36 kg, *p* = 0.335; ADG adjusted LSM: 0.188 vs. 0.157 kg·d^−1^, *p* = 0.203), with only ADG exhibiting a tendency toward difference. Cohen’s d values were 0.43 (final BW) and 0.58 (ADG), indicating a moderate effect size. The feed-to-gain ratio (F:G) of the L-cit group was reduced by 9.38% relative to the CON group, although this difference also failed to reach statistical significance (*p* = 0.087).

### 3.2. Effects of Dietary L-Cit Supplementation on Starter Feed Intake of Lambs

Starter feed intake data collected during the 34 d period from 15 to 48 d of age are summarized in Table 4. The age at first voluntary intake of starter feed differed slightly between groups (CON: 19.8 ± 2.1 d; L-cit: 18.2 ± 1.9 d), exhibiting a tendency toward difference (*p* = 0.092). The average daily feed intake (ADFI, on a DM basis) of starter feed in the L-cit group was significantly increased by 25.96% relative to the CON group (41.6 vs. 52.4 g·d^−1^; *p* = 0.036), with the corresponding 30 d cumulative intake also being significantly elevated (*p* = 0.036).

### 3.3. Effects of Dietary L-Cit Supplementation on Rumen Development of Lambs

The indicators of rumen development measured after slaughter at 48 d of age are presented in Table 5. The fresh rumen weight in the L-cit group was significantly higher than that of the CON group (223.6 vs. 186.4 g; *p* = 0.012), and the rumen-weight-to-live-weight ratio was correspondingly increased (16.92 vs. 15.08 g·kg^−1^; *p* = 0.038). Rumen volume in the L-cit group was elevated by 20.38% compared with the CON group (*p* = 0.009). Morphometric analysis of the ventral sac papillae revealed that papilla length, width, density, and total epithelial thickness were all significantly greater in the L-cit group than in the CON group (all *p* < 0.05), whereas no significant difference in muscle layer thickness was detected between groups (*p* = 0.162). Individual-animal values for all rumen developmental parameters are presented in Supplementary Table S1.

### 3.4. Effects of Dietary L-Cit Supplementation on Ruminal Volatile Fatty Acids of Lambs

Results of the rumen VFA determination are presented in Table 6. The total volatile fatty acid (TVFA) concentration in the rumen of the L-cit group was significantly increased by 19.06% compared with the CON group (64.28 vs. 76.53 mmol·L^−1^; *p* = 0.005). Among the major VFAs, acetate (*p* = 0.019), propionate (*p* = 0.0003), and butyrate (*p* = 0.034) concentrations were all significantly elevated, with propionate exhibiting the most pronounced increase (37.64%; Cohen’s d = 1.83). The remaining VFAs (isobutyrate, isovalerate, valerate, and 2-methylbutyric acid) showed no statistically significant differences between groups (all *p* > 0.05).

With respect to molar proportion composition, the molar proportion of propionate in the L-cit group was significantly increased from 21.41% to 24.75% (*p* = 0.004), while the acetate-to-propionate ratio (A/P) declined from 2.90 to 2.44 (*p* = 0.005), indicating a moderate shift in the rumen fermentation pattern toward propiogenic fermentation. Although the overall fermentation pattern remained acetate-dominant (acetate molar proportion >60% in both groups), the proportional increase in propionate and the corresponding decline in the A/P ratio indicate a meaningful directional shift toward enhanced propiogenic capacity. The A/P ratios in both groups remained above 2.0, within the healthy range, and no risk of subacute ruminal acidosis was observed.

### 3.5. Effects of Dietary L-Cit Supplementation on the Rumen Microbial Community of Lambs

#### 3.5.1. Sequencing Data Quality and α-Diversity

**Figure 1:**
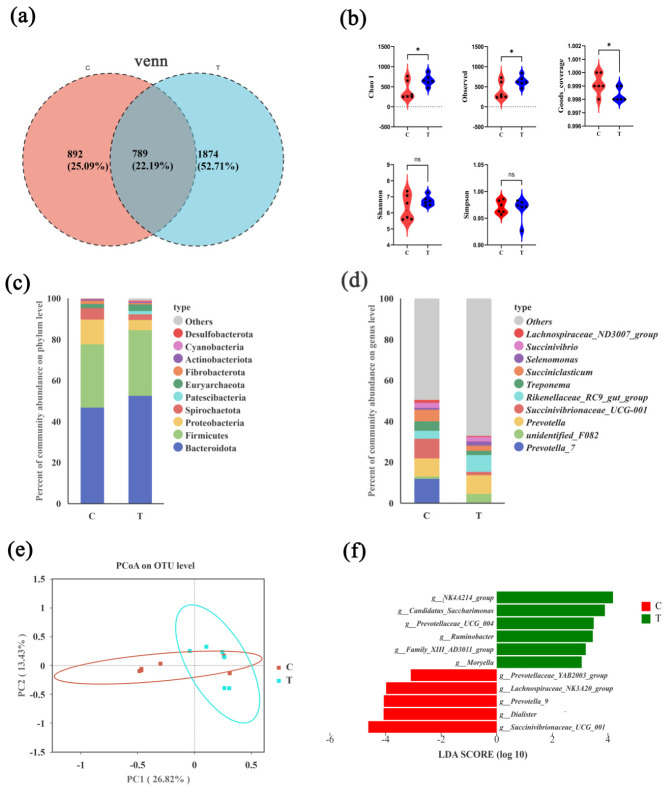
Effects of dietary L-cit supplementation on the rumen microbiome of lambs. (**a**) OTU-based Venn diagram. (**b**) Alpha diversity indices, including Chao 1, Observed, Shannon, Simpson, and Good’s coverage indices. (**c**) Rumen microbial community structure at the phylum level. (**d**) Rumen microbial community structure at the genus level. (**e**) PCoA analysis of the rumen microbiota. (**f**) LDA score plot of OTUs in the rumen microbial community at the genus level. ns indicates no significant difference, * indicates a significant difference (*p* < 0.05). Control group (*n* = 6). L-cit group (*n* = 6).

A total of 1,832,645 effective tags were generated from the 12 rumen fluid samples in the present study, with an average of 91,632 ± 8217 tags per sample and a minimum of 78,436 tags. Good’s coverage was 0.999 ± 0.001 with no significant inter-group difference (*p* = 0.082), indicating that the sequencing depth was sufficient to cover the rumen microbial community. Clustering at 97% sequence similarity yielded 3555 OTUs in total. Venn diagram analysis revealed that 892 OTUs (25.09%) were unique to the CON group, 1874 OTUs (52.71%) were unique to the L-cit group, and 789 OTUs (22.19%) were shared between the two groups (Figure 1a). α-Diversity analysis (Figure 1b) revealed that the Chao1 index (*p* = 0.018) and the Observed species index (*p* = 0.021) of the L-cit group were both significantly higher than those of the CON group, suggesting that L-cit supplementation may have increased species richness in the rumen microbial community. In contrast, no significant differences were observed in the Shannon (*p* = 0.218) or Simpson (*p* = 0.384) indices between groups, indicating that community evenness was not appreciably affected. This pattern (increased richness without altered evenness) is more consistent with an ecological interpretation of increased detection of rare OTUs rather than with an overall enhancement of community diversity.

#### 3.5.2. β-Diversity and Differences in Community Structure

Principal coordinates analysis (PCoA) based on the Bray–Curtis distance matrix (Figure 1e) showed that the CON and L-cit samples exhibited a degree of clustering separation along the PC1 axis (26.82% of variance explained) and the PC2 axis (13.43% of variance explained). PERMANOVA confirmed a significant difference in community composition between the two groups (pseudo-F = 2.17, R^2^ = 0.178, *p* = 0.028, 999 permutations). The PERMDISP test indicated no significant difference in multivariate dispersion between groups (F = 0.54, *p* = 0.487), confirming that the homogeneity-of-dispersion assumption of PERMANOVA was satisfied and that the observed significance reflects a genuine shift in community centroid location rather than differential within-group variability.

At the phylum level (Figure 1c), the dominant phyla in both groups were Bacteroidota, Firmicutes, Proteobacteria, and Spirochaetota, which together accounted for more than 95% of the total community relative abundance. The relative abundances in the CON and L-cit groups were as follows: Bacteroidota, 46.27% vs. 52.76%; Firmicutes, 31.64% vs. 31.72%; Proteobacteria, 11.92% vs. 5.05%; and Spirochaetota, 5.44% vs. 2.81%. Although the relative abundance of Bacteroidota in the L-cit group was elevated by approximately 14.02%, that of Proteobacteria was reduced by 57.63%, and that of Spirochaetota was reduced by 48.35%, no significant differences were detected at the phylum level (all *p* > 0.05).

#### 3.5.3. Genus-Level Microbial Differences and LEfSe Analysis

At the genus level (Figure 1d), the dominant genera comprised Prevotella_7, Prevotella, Succinivibrionaceae_UCG-001 (an uncultured genus within the family Succinivibrionaceae, assigned at the genus rank in SILVA v138.1), Rikenellaceae_RC9_gut_group, and Christensenellaceae_R-7_group. Differential abundance testing demonstrated that the relative abundance of Succinivibrionaceae_UCG-001 in the L-cit group was significantly reduced compared with the CON group (*p* = 0.035), while the relative abundance of Prevotella_7 exhibited a downward tendency (*p* = 0.056), which, according to the a priori definition of the present study (0.05 ≤ *p* < 0.10 indicating a tendency toward difference), was classified as a trend-level change. No other dominant genera differed significantly in relative abundance between groups (all *p* > 0.05).

LEfSe analysis (LDA score > 3.5; Kruskal–Wallis *p* < 0.05; Figure 1f) identified a total of 11 genus-level biomarkers exhibiting significant inter-group differences. The biomarkers enriched in the CON group included *Prevotellaceae_YAB2003_group*, *Lachnospiraceae_NK3A20_group*, *Prevotella_9*, *Dialister*, and *Succinivibrionaceae_UCG-001* (5 in total), whereas those enriched in the L-cit group included *NK4A214_group*, *Candidatus_Saccharimonas*, *Prevotellaceae_UCG-004*, *Ruminobacte*r, *Family_XIII_AD3011_group*, and *Moryella* (6 in total). It should be emphasized that LEfSe is an exploratory analytical tool, and the biomarkers identified above have not yet been validated in independent samples; they should therefore be regarded as candidate targets for subsequent functional studies rather than as definitive conclusions.

### 3.6. Effects of Dietary L-Cit Supplementation on the Rumen Metabolome of Lambs

#### 3.6.1. Overall Metabolite Profile and Multivariate Analysis

**Figure 2:**
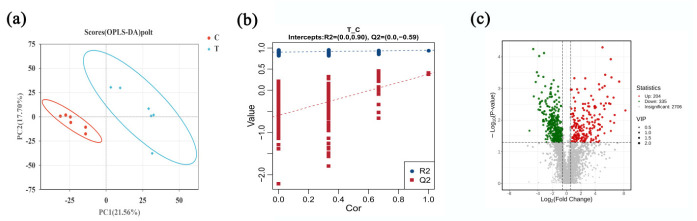
Effects of dietary L-citrulline supplementation on rumen metabolism in lambs. (**a**) Orthogonal partial least squares discriminant analysis (OPLS-DA) score plot of rumen differentially metabolized compounds. PC1 (x-axis) and PC2 (y-axis) represent the first and second principal components, respectively. Dots indicate individual samples, colored by group; ellipses denote 95% confidence regions. (**b**) OPLS-DA permutation test plot. A scatter plot of scores displays the scores of the samples on the first principal component along the x-axis and the scores on the second principal component along the y-axis. When R2Y is greater than Q2Y, the model is considered to have been established. (**c**) Volcano plot of rumen differentially metabolized compounds. The x-axis represents log_2_(fold change), and the y-axis represents −log_10_ (*p*-value). Each data point represents a metabolite: red data points indicate significantly upregulated metabolites (VIP > 1, *p* < 0.05, FC > 1.5), green data points indicate significantly downregulated metabolites (VIP > 1, *p* < 0.05, FC < 0.667), and gray data points indicate metabolites with no significant difference. The size of each data point is proportional to its VIP value. Control group (*n* = 6). L-cit group (*n* = 6).

A total of 3245 metabolites (including positive and negative ion modes) were detected and annotated in the L-cit group and the CON group. The metabolite profiles of these two groups were completely separated by OPLS-DA analysis (Figure 2a). The permutation test results of the OPLS-DA model (Figure 2b) showed that the Q^2^ intercept was negative (Q^2^ < 0), indicating that the model was not overfitted and had good robustness and reliability.

#### 3.6.2. Screening of Differential Metabolites

Using initial screening criteria of VIP > 1, Mann–Whitney U test raw *p* < 0.05, and FC > 1.5 or FC < 0.667, a total of 539 candidate differential metabolites were obtained. Relative to the CON group, 204 metabolites were up-regulated and 335 metabolites were down-regulated in the L-cit group (volcano plot, Figure 2c).

#### 3.6.3. KEGG Pathway Enrichment Analysis

**Figure 3:**
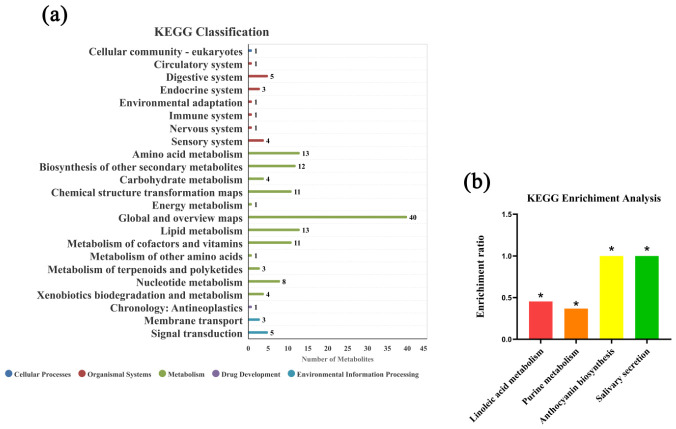
Effects of dietary L-cit supplementation on metabolic pathways in lambs. (**a**) KEGG pathway analysis. The x-axis represents the number of metabolites annotated in the KEGG database. The y-axis represents KEGG pathways. (**b**) KEGG enriched pathway analysis. The x-axis represents metabolic pathways. The y-axis represents the enrichment factor (number of differential metabolites/total number of metabolites in the pathway), indicating the degree of enrichment. * Significant difference (*p* < 0.05).

A total of 147 metabolites were annotated to the KEGG database and assigned to five major functional categories—Metabolism, Organismal Systems, Cellular Processes, Drug Development, and Environmental Information Processing (Figure 3a). Among these, lipid metabolism (13 metabolites), amino acid metabolism (13 metabolites), and metabolism of cofactors and vitamins (11 metabolites) accounted for the largest numbers of annotations. KEGG pathway enrichment analysis (Figure 3b) revealed that four metabolic pathways were significantly enriched among the differential metabolites: linoleic acid metabolism, purine metabolism, anthocyanin biosynthesis, and salivary secretion. These four pathways collectively involved 14 unique differential metabolites with 15 pathway annotations (one metabolite, Guanosine-3′,5′-cGMP, is shared between purine metabolism and salivary secretion; Table 7), of which 3 were up-regulated (cyanidin, keracyanin, and adenine) and 12 were down-regulated in the L-cit group.

### 3.7. Correlation Analysis Between Rumen Microbiota and Differential Metabolites

**Figure 4:**
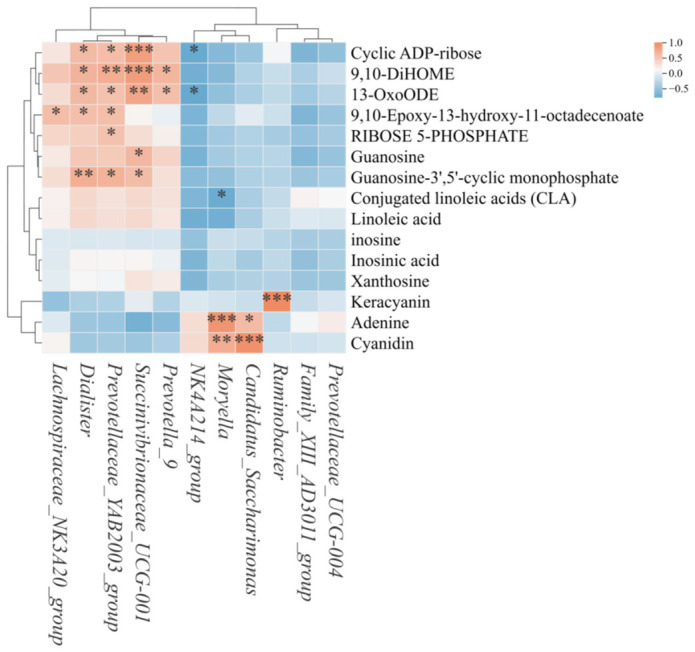
Heatmap showing correlations between the rumen microbiome and rumen metabolites. Red indicates a positive correlation, and blue indicates a negative correlation. * Significant difference (*p* < 0.05), ** Highly significant difference (*p* < 0.01), *** Extremely significant difference (*p* < 0.001).

Following CLR transformation of genus-level relative abundance data, Spearman rank correlation coefficients were used to evaluate the association network between the 11 differentially abundant genera identified by LEfSe and the differential metabolites involved in the 15 enriched KEGG pathway features (Figure 4).

Among the three metabolites up-regulated in the L-cit group, adenine was significantly positively correlated with Moryella (*p* < 0.001) and Candidatus_Saccharimonas (*p* = 0.003); cyanidin was highly significantly positively correlated with both Moryella (*p* = 0.001) and *Candidatus_Saccharimonas* (*p* < 0.001); and keracyanin was highly significantly positively correlated with *Ruminobacte*r (*p* < 0.001). Among the 12 down-regulated metabolites, the products related to linoleic acid metabolism (9,10-DiHOME and 13-OxoODE) were significantly positively correlated with the genera enriched in the CON group (*Lachnospiraceae_NK3A20_group*, *Dialister*, *Prevotellaceae_YAB2003_group*, and *Succinivibrionaceae_UCG-001*; all *p* < 0.05). Likewise, the products related to purine metabolism (guanosine-3′,5′-cGMP and ribose 5-phosphate) were also significantly positively correlated with the same set of CON-enriched genera.

## 4. Discussion

### 4.1. Modulation of Starter Intake and Growth Performance by L-Cit Supplementation

Supplementation of milk replacer with L-cit at 2 g·lamb^−1^·d^−1^ significantly increased the average daily starter intake (ADFI) of male Hu lambs aged 15–45 days from 41.6 ± 9.3 to 52.4 ± 11.7 g DM/d (*p* = 0.036), and the age at first starter intake exhibited an advancing trend (18.2 ± 1.9 vs. 19.8 ± 2.1 d, *p* = 0.092). Average daily gain (ADG) was numerically increased by 20.0% and total weight gain by 16.9%; however, owing to the limited sample size of *n* = 10 per group, neither difference attained statistical significance (*p* > 0.05). Starter intake serves as the upstream driver of substrate supply for early ruminal fermentation and epithelial differentiation [21,22]; accordingly, the marked elevation in ADFI constitutes the starting point for the subsequent ruminal morphological, fermentative, and metabolic responses.

Mechanistically, after absorption across the small-intestinal villi, L-cit bypasses hepatic first-pass metabolism and is converted to L-arginine (L-Arg) in the kidney via argininosuccinate synthase (ASS) and argininosuccinate lyase (ASL); L-Arg subsequently functions as a substrate for the nitric oxide synthase (NOS) and polyamine biosynthetic pathways, participating in the regulation of cellular proliferation and metabolism [5,23,24]. It should be noted, however, that the present study did not measure plasma concentrations of L-cit, L-Arg, or polyamines, nor the expression of pathway-related enzymes (ASS, ASL, NOS, ODC) in rumen epithelial tissue; therefore, whether this metabolic conversion pathway operates at a physiologically relevant scale in suckling lambs remains to be empirically confirmed. In monogastric models, NO regulates gastrointestinal smooth-muscle activity and blood perfusion via the cGMP–PKG pathway, whereas putrescine, spermidine, and spermine upregulate mTORC1 signaling and thereby promote epithelial protein synthesis and proliferation [25]. Against the backdrop of immature rumen–intestinal development in suckling lambs, it is plausible that, the L-cit/L-Arg axis could synergistically advance the morphological and functional maturation of the digestive system, enhancing appetite regulation and palatability recognition of starter feed; although this hypothesis was not directly tested in the present study, the earlier age at first intake and the elevated ADFI are consistent with this interpretation. Gilbreath et al. [7] confirmed that adult ovine rumen microbiota exhibit minimal degradation of L-cit, allowing exogenous L-cit to traverse the rumen largely intact. Although ruminal microbial function remains immature in suckling lambs, the reticular groove reflex during this phase enables milk replacer and its solutes to flow directly into the abomasum via the esophageal groove [21,26], further safeguarding the absorptive efficiency of L-cit in the small intestine and providing a physiological rationale for the dose selection in the present study. It should also be noted that the milk-replacer allowance was recalculated weekly at 2% of fasted BW (DM basis) and administered by gavage; accordingly, the absolute milk-replacer DMI in the L-cit group was marginally higher than in the CON group (173.2 vs. 164.7 g DM/d, +5.15%), tracking the numerical BW advantage, although this difference did not attain statistical significance (*p* = 0.442; Supplementary Table S2). This marginal discrepancy is unlikely to confound the rumen development outcomes, because liquid milk replacer traverses the reticular groove directly to the abomasum and does not supply fermentable substrate to the rumen [21,26]. The significant elevation in starter feed intake (+25.96%, *p* = 0.036) therefore remains the principal dietary factor driving the ruminal morphological, fermentative, and microbial responses reported herein. The intake-promoting and growth-promoting trends observed here are consistent with previously reported findings in piglets [19] and adult sheep [8,27], supporting the application potential of L-cit as a functional amino acid during the early developmental stages of ruminants.

The effective dose identified in the present study (2 g·lamb^−1^·d^−1^, ≈0.22 g·kg^−1^ BW·d^−1^) is internally consistent with the 0.21 g·kg^−1^ BW·d^−1^ effective dose validated in adult Hu ewes by our group [8,9], and falls within the 0.10–0.30 g·kg^−1^ BW·d^−1^ range reported as safe and efficacious in human neonates [18] and neonatal piglets [19]. The convergence across species and developmental stages around this dose range suggests that 0.2–0.3 g·kg^−1^ BW·d^−1^ may represent a generalizable effective dose window for L-cit as an arginine precursor in early-life mammals, with the present study extending this evidence base to suckling ruminants. Importantly, the safety of the selected dose is supported by the absence of any adverse events (diarrhea, reduced feed intake, abnormal behavior, or mortality) throughout the 45 d experimental period. The A/P ratio in the L-citrulline group (2.44) remained well above the subacute ruminal acidosis threshold of 2.0, and branched-chain VFA concentrations were unaltered (all *p* > 0.05), indicating that the dose did not disrupt normal rumen fermentation homeostasis. Nevertheless, the present single-dose design cannot exclude the possibility that alternative doses may yield superior or differential outcomes, and formal dose–response studies in suckling lambs are warranted.

### 4.2. L-Cit Supplementation Promotes Rumen Morphological Development in Suckling Lambs

Rumen morphological development directly governs VFA absorption efficiency, energy utilization, and post-weaning growth performance [1,22]. In the present study, L-cit significantly increased rumen fresh weight at 48 d of age (223.6 ± 31.4 vs. 186.4 ± 28.7 g, *p* = 0.012), the rumen-to-body-weight ratio (16.92 vs. 15.08 g/kg, *p* = 0.038), and rumen volume (821 vs. 682 mL, *p* = 0.009). Ventral-sac papillary length (1742 vs. 1386 μm, *p* = 0.003), width (358 vs. 312 μm, *p* = 0.028), density (67.4 vs. 58.2 papillae/cm^2^, *p* = 0.016), and total epithelial thickness (112.3 vs. 98.4 μm, *p* = 0.033) were all significantly increased, whereas no significant difference was observed in muscular layer thickness (*p* = 0.162), indicating that the action of L-cit specifically targets the epithelium rather than the muscular layer. These metrics fall within the ranges reported for suckling lambs and calves by Lin et al. [10], Sun et al. [13], and Khan et al. [21] (papillary length 900–2000 μm, width 250–440 μm, rumen-to-body-weight ratio 14–18 g/kg), with the L-cit group approaching the upper bounds of these ranges. Whereas previous L-cit studies in ruminants have focused predominantly on production performance, blood biochemistry, or microbial composition [8,27], the present quantitative morphometric data suggest that L-cit supplementation was associated with enhanced rumen epithelial development during the suckling phase.

Mechanistically, two synergistic pathways may account for these findings, although neither was directly assessed at the molecular level in the present study. First, in monogastric neonatal models, after conversion to L-Arg derived from L-cit conversion in the kidney and peripheral tissues is processed by ornithine decarboxylase (ODC) to yield putrescine, which is further synthesized into spermidine and spermine. These polyamines stabilize nucleic acid structure, modulate eIF5A modification, and activate mTORC1, thereby driving the G1/S-phase transition and protein synthesis in epithelial cells [23,25]. Whether this polyamine–mTOR cascade is activated in the rumen epithelium of L-cit-supplemented suckling lambs awaits verification through measurements of epithelial polyamine concentrations and the phosphorylation status of mTOR pathway components (e.g., p-mTOR, p-S6K1, p-4E-BP1). Arginine has been shown to activate the mTOR–S6K1 signaling axis and promote cell proliferation in neonatal pigs [28], and Connor et al. [29] reported that analogous molecular regulators, including components of the IGF and MAPK pathways, are involved in rumen epithelial development during weaning. Second, as shown in Section 4.4, ruminal butyrate concentration in the L-cit group was significantly elevated (8.91 vs. 7.42 mmol/L, *p* = 0.034). Butyrate is the principal energy substrate for the rumen epithelium and a ligand for GPR41/43 signaling; through inhibition of histone deacetylases (HDAC), it upregulates cyclin D1 and IGF-1R expression, thereby promoting papillary elongation and epithelial thickening [30,31,32]. The hypothesized synergy between the polyamine–mTOR and butyrate–HDAC pathways would provide a coherent and self-consistent explanation for papillary elongation, epithelial thickening, and increased absolute rumen weight, thereby connecting the elevated ADFI reported in Section 4.1 with the elevated butyrate concentrations discussed in Section 4.4. However, in the absence of direct measurements of plasma polyamine levels, rumen epithelial mTOR phosphorylation, or HDAC activity, this integrated mechanistic model remains a hypothesis informed by the existing literature rather than a conclusion established by the present data.

The implications of early rumen morphological development extend beyond the suckling phase itself. Yáñez-Ruiz et al. [33] defined the period from birth to weaning (0–8 weeks) as the “programming window,” during which the rumen microbiota and epithelium exhibit the greatest plasticity; nutritional interventions during this phase can exert long-lasting effects on feed efficiency, lean tissue accretion, and metabolic health by altering developmental trajectories. The concurrent increases in papillary length, density, and epithelial thickness observed in the L-cit group imply enhanced VFA absorptive capacity per unit rumen surface area; this structural advantage is anticipated to persist through the weaning transition and finishing periods, ultimately translating into improved feed conversion efficiency [1,21].

### 4.3. L-Cit Remodels Rumen Bacterial Community Structure and Functional Potential

The shaping effect of L-cit on the rumen microbiota is reflected first in α-diversity. The Chao1 and observed-species indices were significantly elevated in the L-cit group, whereas the Shannon and Simpson indices showed no corresponding change, indicating that the increase in species richness arose from the expansion of rare taxa rather than from alterations in the evenness of dominant groups. Ecologically, the expansion of rare taxa frequently signals an increase in functional redundancy, enabling fermentation homeostasis to be maintained under substrate fluctuations [34,35]. As an L-Arg precursor, L-cit has been reported to be absorbed by the rumen epithelium and enter the arginine–NO–polyamine axis [36,37] while also being utilized as a nitrogen source by ruminal microbes [7]; if this dual role is operative in suckling lambs—a possibility that was not verified by circulating or tissue-level indicators in the present study—it could provide a growth window for rare taxa with diverse nitrogen-metabolic requirements, thereby expanding the community niche.

At the phylum level, the relative abundance of Bacteroidota in the L-cit group exhibited a numerical increase of 14.02%, whereas Proteobacteria decreased by 57.63%; although these changes did not reach statistical significance (*p* > 0.05), they are consistent with the directional succession of the rumen microbiota during maturation. Bacteroidota are central executors of soluble polysaccharide and non-fibrous carbohydrate metabolism in the rumen, and their elevation is frequently accompanied by increased CAZyme-encoding potential and activation of propionate-generating pathways [16,38]. A high relative abundance of Proteobacteria in young ruminants is generally regarded as a marker of ecological imbalance [34,39]; their decline in the L-cit group is consistent with a shift toward a more anaerobic, fermentation-competent community. The L-Arg–NO pathway, through its documented effects on mucosal oxygen tension [23], has been proposed as a plausible mechanism for this indirect microbiota remodeling; however, this interpretation is speculative in the present context, as neither mucosal NO concentrations nor oxygen tension were measured.

By contrast, differences at the genus level carry greater functional significance. Notably, the abundance of Succinivibrionaceae_UCG-001 was significantly reduced (*p* = 0.035). Members of this family generate succinate via the fumarate reductase pathway, which is subsequently decarboxylated by downstream microbiota and converted to propionate [39,40]. According to the classical interpretation, a reduction in this group would be expected to lower propionate output; however, Section 4.4 demonstrates that propionate concentration and molar proportion in the L-cit group were concurrently and significantly elevated. The mechanistic resolution of this discrepancy, involving pathway-level compensation by the L-cit-enriched genera, is detailed in Section 4.4.

The marker genera identified by LEfSe in the L-cit group support the foregoing hypothesis. *Prevotellaceae_UCG-004* possesses propionate-generating capacity through both the succinate and acrylate pathways and constitutes a hub of non-fibrous carbohydrate metabolism in the rumen [41]. *Ruminobacter* is a typical fiber-degrading and starch-hydrolyzing genus whose abundance correlates positively with nitrogen utilization efficiency [42], and its enrichment may be directly attributable to the available nitrogen source supplied by L-cit. The *NK4A214_group* (family Oscillospiraceae) is associated with fiber degradation and butyrate-precursor production, with its proportion increasing in well-developed young ruminants [43]. *Candidatus_Saccharimonas* (phylum TM7) has recently been reported to be associated with host immune homeostasis [44]; however, its function in the rumen still requires targeted mechanistic investigation, and we therefore designate it as a candidate regulatory node rather than a confirmed regulator. *Moryella* was reported to exhibit proinflammatory potential in a bovine mastitis model [45], but the function of this genus in the ruminant rumen has not been systematically characterized; its enrichment should not be equated simplistically with pathogenic risk, and a more conservative interpretation is that it reflects an overall alteration in the ruminal nitrogen-metabolic microenvironment. Taken together, the L-cit-associated microbiota can be summarized as a compositional shift favoring genera with diverse propionate-generating capacity, the fermentation implications of which are examined in Section 4.4.

### 4.4. L-Cit Enhances Propionate Proportion in Ruminal Fermentation and Its Implications for Energy Metabolism

Concomitant with the alterations in microbial community structure, L-cit exerted systemic regulatory effects on the rumen VFA profile. Compared with the CON group, the total VFA (TVFA) concentration in the L-cit group increased significantly from 64.28 to 76.53 mmol/L (*p* = 0.005), with significant elevations in acetate (+15.8%, *p* = 0.019), propionate (+37.6%, *p* < 0.001), and butyrate (+20.1%, *p* = 0.034). The molar proportion of propionate increased from 21.41% to 24.75% (*p* = 0.004), and the acetate-to-propionate (A/P) ratio decreased from 2.90 to 2.44 (*p* = 0.005), indicating a significant directional shift toward enhanced propiogenic fermentation capacity, although the overall fermentation profile remained acetate-dominant, as reflected by the A/P ratio remaining above 2.0 and the acetate molar proportion exceeding 60% in both groups.

The significant decrease in *Succinivibrionaceae_UCG-001* alongside the significant increase in propionate flux constitutes an apparent paradox whose resolution lies in the functional redundancy of propionate-generating pathways. Rumen propionate is produced primarily through two routes: the succinate pathway (mediated by Succinivibrionaceae, Prevotellaceae, Selenomonas, etc.) and the acrylate pathway (mediated principally by Megasphaera and certain Prevotella groups) [46]. *Prevotellaceae_UCG-004*, *Ruminobacter*, and the *NK4A214_group* enriched in the L-cit group all possess metabolic potential to generate propionate via the succinate pathway or by direct fermentation [40,42], and their collective contribution to net propionate yield exceeded the flux loss attributable to the reduction in *Succinivibrionaceae_UCG-001*. The regulatory action of L-cit is therefore achieved not by amplifying the metabolic activity of a single genus but by reallocating metabolic pathways at the community scale, thereby effecting a shift in fermentation pattern.

From the perspective of host energy metabolism, the coordinated rise in propionate carries explicit physiological significance. Approximately 50–60% of glucose in ruminants is derived from hepatic gluconeogenesis using propionate as the precursor [47,48]; as suckling lambs initiate starter intake and ruminal fermentation capacity is established, the importance of propionate as a gluconeogenic precursor increases markedly [1]. In the present study, the L-cit group exhibited elevated propionate, an A/P ratio reduced to 2.44 (still above the SARA warning threshold of 2.0), and increased TVFA, collectively constituting an acetate-dominant fermentation environment with significantly enhanced propiogenic capacity, maintained within a healthy homeostatic range (A/P > 2.0). These changes are metabolically corroborated by the 20% numerical improvement in ADG and the significant increase in ADFI reported in Section 4.1; that is, the increased propionate furnishes a more abundant supply of glucose precursors, potentially providing the material basis underlying the observed growth-promoting trend. The direction of these findings is consistent with previous reports from our group on L-cit-mediated regulation of VFA proportions in adult Hu sheep [8] and extends this effect to the suckling phase, confirming developmental-stage specificity for the VFA-modulating action of L-cit.

The significant increase in butyrate (7.42 → 8.91 mmol/L, *p* = 0.034) directly links the fermentation alterations to the rumen epithelial morphological development discussed in Section 4.2. Butyrate is the preferred energy substrate for the rumen epithelium and a key inducer of papillary development; it can expand papillary length and absorptive surface area through the IGF-1/IGFBP3 and MAPK pathways [29,34]. The four indicators—elevated butyrate, papillary elongation, epithelial thickening, and increased absolute rumen weight—are mechanistically self-consistent and link fermentation output to the epithelial development reported in Section 4.2. Elevated butyrate activates FFAR2/3-mediated proliferative signaling, while the polyamine–mTOR axis described earlier (Section 4.2) operates synergistically; together, these pathways account for the observed papillary and epithelial responses. No significant changes were observed in branched-chain or other short-chain VFAs such as isobutyrate or valerate, indicating that the action of L-cit is relatively specific to mainstream fermentation pathways rather than constituting a broad perturbation of the rumen fermentation profile. Collectively, the VFA, morphological, and microbiological data converge on a consistent interpretation: the microbial compositional shifts described in Section 4.3 underpin the propionate-predominant fermentation pattern, which in turn supplies both the gluconeogenic precursors and the epithelial energy substrates that account for the developmental outcomes reported in Section 4.2.

### 4.5. L-Cit Modulates the Rumen Metabolome and Affects Key Metabolic Pathways

Untargeted metabolomics further revealed the systemic regulatory effects of L-cit on the rumen metabolome. With OPLS-DA model support (R^2^Y = 0.90; permutation-test Q^2^ intercept = −0.63, indicating no overfitting), 539 differential metabolites were identified, and KEGG enrichment analysis pinpointed four pathways: linoleic acid metabolism, purine metabolism, anthocyanin biosynthesis, and salivary secretion. The biological plausibility of these four pathways differs markedly, however, and warrants separate evaluation.

Among them, the alterations in the linoleic acid metabolism pathway possess the most robust biological foundation. Linoleic acid (LA), conjugated linoleic acid (CLA), 9,10-DiHOME, and 13-OxoODE were all significantly downregulated in the L-cit group. CLA and LA are key intermediates in the trans-fatty-acid biohydrogenation occurring in the rumen, and their abundance is jointly regulated by the activity of biohydrogenating microbes and substrate flux [49,50]. At elevated concentrations, LA and its oxidative derivatives (e.g., 9,10-DiHOME, 13-HPODE) exert growth-inhibitory effects on Gram-positive ruminal bacteria, particularly fiber-degrading and propionate-producing bacteria [51,52]. Accordingly, the systemic downregulation of LA and its derivatives in the L-cit group may originate from accelerated rumen biohydrogenation driven by the elevated ADFI, establishing a more favorable microenvironment for normal fermentation microbiota. This is consistent with the net increases in TVFA and propionate reported in Section 4.4 and constitutes a lipid-metabolic hub linking functional amino acid action, ruminal lipid metabolism, and fermentation regulation.

The purine metabolism pathway likewise carries clear biological significance. Adenine was significantly upregulated, whereas xanthosine, guanosine, inosine, inosinic acid, and ribose-5-phosphate were significantly downregulated, suggesting that purine metabolism shifted away from synthesis and salvage and toward suppression of degradation. Adenine has been identified as an anti-inflammatory metabolite in M-CSF–differentiated macrophages and can attenuate LPS-induced NF-κB pathway activation, thereby reducing the production of proinflammatory cytokines and inflammatory lipid mediators [53,54]. Xanthosine is a substrate of xanthine oxidase, and its decline may reduce superoxide-anion generation through this enzymatic route, indirectly mitigating oxidative stress [55]. Purine derivatives are also key intermediates in microbial nitrogen metabolism and nucleic acid turnover in the rumen, and their profile shifts may reflect alterations in the balance between microbial nucleotide synthesis and degradation arising from the available nitrogen source provided by L-cit [56]. These changes point to potential antioxidant and anti-inflammatory directions; however, because the present study did not directly measure targeted indices such as serum or rumen-local SOD, GSH-Px, T-AOC, MDA, IL-6, or TNF-α, the specific effects await further validation.

The anthocyanin biosynthesis and salivary secretion pathways require more critical interpretation. Anthocyanin biosynthesis is a plant secondary metabolic pathway dependent on plant-specific enzyme systems including CHS, F3H, DFR, and ANS; neither ruminant hosts nor rumen microorganisms possess complete biosynthetic capability [57]. The cyanidin and keracyanin detected in the rumen are more plausibly derived from plant feed residues, including those of corn (whose seed coat and aleurone layer contain small amounts of anthocyanins and their glycosides) and soybean meal [58,59]; the significantly higher ADFI in the L-cit group than in the CON group (52.4 vs. 41.6 g DM/d, *p* = 0.036) is the direct driver of the difference in plant-derived anthocyanin residues within the rumen. The salivary secretion pathway is intrinsically a host-organ-specific pathway; its enrichment signal in the rumen-content metabolome more likely reflects the preferential KEGG-database categorization of cross-pathway shared metabolites such as cGMP and cADP-ribose into this pathway [60] rather than the actual execution of salivary secretion functions by rumen tissue.

### 4.6. Rumen Microbiota–Metabolite Association Network and Its Potential Regulatory Implications

Building on the synchronous dynamic changes in microbiota and metabolites, the present study evaluated their associations through Spearman rank correlation analysis and identified three groups of significant associations with clear biological significance.

The first group involves significant positive correlations of *Candidatus_Saccharimonas* with adenine and cyanidin. *Candidatus_Saccharimonas* (phylum Saccharibacteria/TM7) comprises ultra-small bacteria with highly streamlined genomes; previous studies have suggested their potential involvement in regulating host immune homeostasis through epibiotic surface attachment [45,61]. The covariance of this genus with adenine may reflect its node-level role in the purine turnover network; however, the precise functional contributions still require targeted resolution at the metagenomic and metatranscriptomic levels [62].

The second group is the highly significant positive correlation between *Ruminobacter* and keracyanin. *Ruminobacter* is a typical starch-hydrolyzing and fiber-degrading genus in the rumen, and its abundance is closely associated with feed-particle retention time and carbohydrate metabolic flux [43,63]. This association more likely reflects the covariance of *Ruminobacter* with feed-derived metabolites (including plant-derived anthocyanin residues) under active feeding states rather than a direct metabolic causal chain.

The third group consists of positive correlations of Moryella with adenine and cyanidin. Guo et al. [45] reported potential associations of *Moryella* with rumen LPS levels and systemic inflammation in a bovine mastitis model; however, the metabolic functions and host interactions of different species within the genus *Moryella* exhibit considerable heterogeneity, and the core role of this genus in the rumen has yet to be systematically resolved through multidimensional evidence from functional genomics, metatranscriptomics, or culturomics. Changes in *Moryella* abundance more likely reflect the overall alteration of the ruminal nitrogen-metabolic microenvironment in the L-cit group, and the health implications of these changes can be determined only in conjunction with host indices such as serum inflammatory cytokines and rumen epithelial tight-junction protein expression.

### 4.7. Limitations and Future Perspectives

Several limitations of the present study should be acknowledged. First, although rumen weight, rumen volume, papillary morphology, epithelial thickness, VFA profiles, microbial community structure, and rumen metabolomic profiles were evaluated, no direct functional measurements of epithelial barrier integrity were performed. For example, tight-junction proteins, including ZO-1, occludin, claudin family members, and E-cadherin, were not quantified, and epithelial permeability or electrophysiological function was not assessed using methods such as FITC-dextran permeability, transepithelial electrical resistance, or Ussing chamber assays. Therefore, the increased epithelial thickness and improved papillary morphology observed in the L-cit group should be interpreted as evidence of enhanced rumen morphological development rather than definitive evidence of improved epithelial barrier function.

Second, although the metabolomic results indicated changes in purine metabolism and linoleic acid metabolism, which may be related to oxidative and inflammatory pathways, the present study did not directly measure oxidative stress or inflammatory status. Biomarkers such as SOD, GSH-Px, CAT, T-AOC, MDA, ROS, IL-1β, IL-6, TNF-α, IL-10, LPS, and NF-κB-related signaling molecules were not determined in plasma, rumen fluid, or rumen epithelial tissue. Consequently, any potential antioxidant or anti-inflammatory implications of L-cit supplementation should be considered hypothesis-generating and require further experimental validation.

Third, sample size and statistical power considerations warrant transparent discussion. The post-mortem analyses were performed using six lambs per group, and in vivo growth performance was measured on ten lambs per group. Retrospective power analyses (G*Power 3.1.9.7; α = 0.05, two-tailed independent-samples t-test) revealed that for the primary rumen developmental and VFA endpoints, the observed effect sizes were large (Cohen’s d = 1.04–1.84), with achieved power ranging from 0.38 to 0.82 and 15 of 17 endpoints reaching statistical significance (*p* < 0.05), confirming that *n* = 6 per group was adequate for these primary outcomes, consistent with comparable multi-omics rumen development studies [10,13,34]. By contrast, growth performance endpoints exhibited moderate effect sizes (Cohen’s d = 0.43–0.79) and low achieved power (0.14–0.35), indicating that the study was underpowered for these secondary outcomes. The 20% numerical increase in ADG (Cohen’s d = 0.58) would require approximately 38 lambs per group to achieve 80% power—a sample size rarely feasible in neonatal lamb studies incorporating intensive post-mortem and multi-omics analyses. To mitigate the risk of over-interpretation, we have reported Cohen’s d values and described non-significant growth results as numerical trends with moderate effect sizes warranting confirmation in adequately powered trials. Future confirmatory studies targeting growth performance as a primary endpoint should enroll at least 30–40 lambs per group, whereas rumen developmental endpoints can be adequately powered with *n* = 8–10 per group (based on d = 1.2, power = 0.80); the complete retrospective power analysis is provided in Supplementary Table S3. Additionally, only one supplementation dose and one terminal sampling time point were evaluated, which limits the ability to establish dose–response relationships, temporal dynamics, or long-term effects during and after weaning. Moreover, only male Hu lambs were used; therefore, the applicability of these findings to female lambs, other breeds, or different management systems remains to be determined.

Fourth, 16S rRNA sequencing and untargeted metabolomics provide association-level evidence but cannot establish causality among L-cit supplementation, microbial taxa, rumen metabolites, and host epithelial responses. Future studies should integrate metagenomics, metatranscriptomics, targeted metabolite validation, epithelial transcriptomics or proteomics, and direct functional assays of barrier integrity, oxidative stress, and inflammatory responses. Such studies will be necessary to clarify whether L-cit directly improves rumen epithelial function and host health, beyond its effects on rumen morphology, fermentation, and microbiota–metabolome profiles.

Fifth, and most critically in relation to the proposed mechanistic framework, the present study did not measure any direct indicators of the arginine–NO–polyamine pathway that forms the basis of the mechanistic discussion in Section 4.1, Section 4.2 and Section 4.3. Specifically, plasma concentrations of L-citrulline, L-arginine, L-ornithine, and polyamines (putrescine, spermidine, and spermine) were not determined; nitric oxide metabolites (nitrate + nitrite) were not quantified; and the expression of key enzymes and signaling molecules in the pathway—including argininosuccinate synthase (ASS1), argininosuccinate lyase (ASL), nitric oxide synthase (NOS), ornithine decarboxylase (ODC1), and the mTOR–S6K1 signaling axis—was not assessed in rumen epithelial tissue. As a consequence, all references to the arginine–NO–polyamine pathway in the present Discussion should be interpreted strictly as literature-informed hypotheses that provide a plausible but unverified interpretive framework for the observed phenotypic outcomes. Direct validation of this mechanistic model in suckling lambs will require future studies incorporating targeted plasma amino acid and polyamine profiling (e.g., LC-MS/MS), quantitative analysis of pathway gene/protein expression in fresh-frozen rumen epithelial tissue (RT-qPCR and/or Western blot), and functional epithelial assessments (Ussing chamber or FITC-dextran permeability). These studies are currently being planned by our research group.

## 5. Conclusions

Dietary L-citrulline at 2 g·lamb^−1^·d^−1^ enhanced starter feed intake and promoted rumen epithelial morphological development in suckling Hu lambs, as indicated by increased papillary length, papillary density, and epithelial thickness. L-cit supplementation also shifted ruminal fermentation toward a propionate-predominant pattern, increased microbial richness, enriched several genera associated with carbohydrate fermentation, and altered rumen metabolites mainly involved in linoleic acid and purine metabolism. These findings provide preliminary morphological, fermentation, microbiome, and metabolomic evidence supporting L-citrulline as a candidate functional feed additive during the suckling phase. However, because neither host-level indicators of the arginine–NO–polyamine pathway (plasma arginine, citrulline, polyamines, and epithelial gene expression) nor direct measurements of epithelial barrier integrity, oxidative stress, or inflammatory markers were obtained, the mechanistic interpretation linking L-citrulline to rumen development via this pathway remains hypothesis-generating. Future mechanism-based studies incorporating targeted metabolomic profiling, epithelial molecular analyses, and functional assays are required to validate the proposed pathway and to confirm the effects of L-citrulline on rumen barrier function and host health. In addition, the practical efficacy of this supplementation strategy should be validated in larger-scale production studies evaluating growth performance, feed efficiency, health status, weaning transition, and economic outcomes.

## Figures and Tables

**Table 1:** Ingredient composition and nutrient levels of the milk replacer.

Items	Content (%)
Milk replacer ingredients	
Whole milk powder	65.00
High-protein whey powder	18.00
Whey protein concentrate (WPC)	2.00
Soy protein concentrate	10.00
L-Lysine	2.20
DL-Methionine	0.80
L-Threonine	1.00
NaCl	0.50
Trace mineral premix ^1^	0.25
Vitamin premix ^2^	0.25
Total	100.00
Nutrient level ^3^	
ME (MJ·kg^−1^)	18.73
DM (%)	94.11
CP (%)	23.56
EE (%)	12.35
CF (%)	2.18
Ash (%)	8.33
Ca (%)	1.16
P (%)	0.84

Notes: ^1^ The trace mineral premix supplied per kilogram of milk replacer: Cu 8 mg, Fe 60 mg, Zn 80 mg, Mn 40 mg, I 1 mg, Se 0.5 mg, Co 0.8 mg. ^2^ The vitamin premix supplied per kilogram of milk replacer: vitamin A 25,000 IU, vitamin D_3_ 5200 IU, vitamin E 40 IU. ^3^ ME values were calculated, whereas the other nutrients were measured (*n* = 3 replicate analyses).

**Table 2:** Ingredient composition and nutrient levels of the starter feed.

Items	Content (%)
Starter feed ingredients	
Corn	62.80
Soybean meal	26.20
Wheat bran	7.00
CaHPO_4_	1.00
Limestone	1.50
NaCl	0.50
Premix ^1^	1.00
Total	100.00
Nutrient level ^2^	
ME (MJ·kg^−1^)	12.83
DM (%)	83.16
CP (%)	18.67
EE (%)	3.42
CF (%)	8.69
Ash (%)	8.78
Ca (%)	1.46
P (%)	0.72

Notes: ^1^ The premix supplied per kilogram of starter feed: Cu 10 mg, Fe 80 mg, Zn 100 mg, Mn 50 mg, I 0.8 mg, Se 0.3 mg, Co 0.5 mg, vitamin A 15,000 IU, vitamin D_3_ 3000 IU, vitamin E 50 IU. ^2^ ME values were calculated, whereas the other nutrients were measured (*n* = 3 replicate analyses).

**Table 3:** Effects of dietary L-cit supplementation on the growth performance of suckling lambs.

Items	CON (*n* = 10)	L-Cit (*n* = 10)	SEM	*p*-Value	Cohen’s d
Initial BW (kg)	5.19 ± 0.96	5.29 ± 0.69	0.26	0.787	0.12
Final BW (kg)	12.36 ± 1.84	13.21 ± 2.05	0.62	0.335	0.43
Total weight gain, TWG (kg)	7.17 ± 1.63	8.38 ± 1.84	0.55	0.141	0.69
Average daily gain, ADG (g·d^−1^)	157 ± 34	188 ± 40	12	0.203	0.58
Feed-to-gain ratio, F:G (DMI/gain)	1.92 ± 0.24	1.74 ± 0.21	0.07	0.087	0.79

Note: Data are presented as mean ± SD. SEM denotes the standard error of the mean adjusted by ANCOVA; *p*-values were corrected for the initial BW covariate.

**Table 4:** Effects of dietary L-cit supplementation on starter feed intake of suckling lambs.

Items	CON (*n* = 10)	L-Cit (*n* = 10)	*p*-Value
Age at first intake (d)	19.8 ± 2.1	18.2 ± 1.9	0.092
Average daily feed intake, ADFI (g DM·d^−1^)	41.6 ± 9.3	52.4 ± 11.7	0.036
Total intake (g DM, 15–48 d)	1248 ± 279	1572 ± 351	0.036

**Table 5:** Effects of dietary L-cit supplementation on rumen development of suckling lambs.

Items	CON (*n* = 6)	L-Cit (*n* = 6)	*p*-Value
BW at slaughter (kg)	12.36 ± 1.84	13.21 ± 2.05	0.335
Rumen weight (g)	186.4 ± 28.7	223.6 ± 31.4	0.012
Rumen weight/BW (g·kg^−1^)	15.08 ± 1.92	16.92 ± 1.78	0.038
Rumen volume (mL)	682 ± 98	821 ± 112	0.009
Papillae length (μm)	1386 ± 214	1742 ± 246	0.003
Papillae width (μm)	312 ± 41	358 ± 47	0.028
Papillae density (n·cm^−2^)	58.2 ± 7.1	67.4 ± 8.3	0.016
Epithelial thickness (μm)	98.4 ± 12.6	112.3 ± 14.1	0.033
Muscle layer thickness (μm)	486 ± 71	534 ± 78	0.162

Note: Total epithelial thickness includes the stratum corneum, stratum granulosum, stratum spinosum, and stratum basale; ventral sac sampling was performed at the central region of the rumen ventral sac; for each lamb, at least 10 intact papillae were measured and averaged.

**Table 6:** Effects of dietary L-cit supplementation on rumen volatile fatty acids of suckling lambs.

Items	CON (*n* = 6)	L-Cit (*n* = 6)	*p*-Value
Concentration (mmol·L^−1^)			
Total VFA (TVFA)	64.28 ± 8.42	76.53 ± 9.17	0.005
Acetate (C_2_)	39.84 ± 5.31	46.12 ± 5.68	0.019
Propionate (C_3_)	13.76 ± 2.48	18.94 ± 3.12	0.0003
Butyrate (C_4_)	7.42 ± 1.36	8.91 ± 1.54	0.034
Isobutyrate	0.86 ± 0.18	0.79 ± 0.16	0.368
Valerate	1.12 ± 0.24	1.28 ± 0.27	0.178
Isovalerate	0.94 ± 0.21	0.98 ± 0.23	0.689
2-Methylbutyric acid	0.34 ± 0.08	0.41 ± 0.09	0.082
Molar proportion (mol %)			
Acetate	61.98 ± 2.74	60.26 ± 2.58	0.161
Propionate	21.41 ± 2.13	24.75 ± 2.46	0.004
Butyrate	11.54 ± 1.38	11.64 ± 1.47	0.878
A/P ratio	2.90 ± 0.36	2.44 ± 0.29	0.005

**Table 7:** Fifteen ruminal differential metabolites involved in the four significantly enriched KEGG pathways.

Metabolite	VIP	Log_2_FC	Up/Down	*p*-Value	MSI Level
9,10-DiHOME	1.400	−1.390	Down	0.041	Level 2
9,10-Epoxy-13-hydroxy-11-octadecenoate	1.433	−1.129	Down	0.033	Level 2
13-OxoODE	1.671	−1.312	Down	0.008	Level 2
Conjugated linoleic acid (CLA)	1.461	−1.287	Down	0.044	Level 1
Linoleic acid	1.631	−1.126	Down	0.020	Level 1
Guanosine	1.479	−2.316	Down	0.023	Level 1
Adenine	1.494	+1.525	Up	0.023	Level 1
Guanosine-3′,5′-cGMP	1.594	−2.279	Down	0.017	Level 2
Ribose 5-phosphate	1.763	−2.672	Down	0.004	Level 2
Xanthosine	1.857	−2.363	Down	0.002	Level 2
Inosine	1.719	−3.108	Down	0.005	Level 1
Inosinic acid (IMP)	1.500	−2.361	Down	0.023	Level 2
Cyanidin	1.979	+2.575	Up	0.001	Level 2
Keracyanin	2.012	+5.006	Up	0.001	Level 2
Guanosine-3′,5′-cGMP ^1^	1.594	−2.279	Down	0.017	Level 2
Cyclic ADP-ribose	1.676	−1.974	Down	0.013	Level 2

Note: ^1^ Guanosine-3′,5′-cGMP is concurrently annotated to both the purine metabolism and salivary secretion pathways in the KEGG database owing to its role as a shared second messenger; it is listed once in this table to avoid duplication.

## Data Availability

The 16S rRNA gene sequencing raw reads generated in this study have been deposited in the NCBI Sequence Read Archive (SRA) under BioProject accession number PRJNA1460896 and are publicly accessible at https://www.ncbi.nlm.nih.gov/bioproject/?term=PRJNA1460896 (accessed on 30 May 2026). The LC-MS untargeted metabolomics dataset has been deposited in the OMIX database of the National Genomics Data Center (NGDC), China National Center for Bioinformation (CNCB)/Beijing Institute of Genomics, Chinese Academy of Sciences, under the accession number OMIX017048, and is publicly accessible at https://ngdc.cncb.ac.cn/omix/release/OMIX017048 (accessed on 30 May 2026). The remaining datasets generated and/or analyzed during the current study—including individual-animal phenotypic records, rumen volatile fatty acid raw chromatograms, and histomorphometric measurements—are available from the corresponding author upon reasonable request.

## Supplementary Materials

The following supporting information can be downloaded at: https://www.mdpi.com/article/10.3390/ani16111728/s1, Table S1: Individual-animal values of rumen developmental parameters in suckling Hu lambs; Table S2: Milk-replacer dry-matter intake of suckling Hu lambs during the experimental period; Table S3: Retrospective (observed) statistical power analysis for the primary growth, rumen developmental, and ruminal fermentation endpoints.

## Abbreviations

The following abbreviations are used in this manuscript:

ADG	Average Daily Gain
ADFI	Average Daily Starter Intake
Ash	Crude Ash
ASL	Argininosuccinate Lyase
ASS	Argininosuccinate Synthase
BW	Body Weight
CF	Crude Fiber
CLA	Conjugated Linoleic Acid
CP	Crude Protein
DM	Dry Matter
DMI	Dry Matter Intake
EE	Ether Extract
FC	Fold Change
HDAC	Histone Deacetylases
HMDB	Human Metabolomics Database
KEGG	Kyoto Encyclopedia of Genes and Genomes
LA	Linoleic Acid
LC-MS	Liquid Chromatography–Mass Spectrometry Coupling
LDA	Linear Discriminant Analysis
LEfSe	Effect Size of Linear Discriminant Analysis
NOS	Nitric Oxide Synthase
PCA	Principal Component Analysis
PCoA	Principal Coordinate Analysis
OPLS-DA	Orthogonal Partial Least Squares Discriminant Analysis
QC	Quality Control
TVFA	Total Volatile Fatty Acid
VFA	Volatile Fatty Acid
VIP	Variable Importance in Projection
